# A short-term rodent model for non-alcoholic steatohepatitis induced by a high-fat diet and carbon tetrachloride

**DOI:** 10.1042/BSR20231532

**Published:** 2024-05-08

**Authors:** Layanne C.C. Araujo, Carolina C.B. Dias, Felipe G. Sucupira, Leandra N.Z. Ramalho, João Paulo Camporez

**Affiliations:** 1 Department of Physiology, Ribeirao Preto School of Medicine, University of Sao Paulo, Brazil; 2 Department of Pathology and Legal Medicine, Ribeirao Preto School of Medicine, University of Sao Paulo, Brazil

**Keywords:** carbon tetrachloride, High-fat diet, non-alcoholic steatohepatitis

## Abstract

Several models of mice-fed high-fat diets have been used to trigger non-alcoholic steatohepatitis and some chemical substances, such as carbon tetrachloride. The present study aimed to evaluate the joint action of a high-fat diet and CCl_4_ in developing a short-term non-alcoholic steatohepatitis model. C57BL6/J mice were divided into two groups: standard diet-fed (SD), the high-fat diet-fed (HFD) and HFD + fructose-fed and carbon tetrachloride (HFD+CCl_4_). The animals fed with HFD+CCl_4_ presented increased lipid deposition compared with both SD and HFD mice. Plasma cholesterol was increased in animals from the HFD+CCl_4_ group compared with the SD and HFD groups, without significant differences between the SD and HFD groups. Plasma triglycerides showed no significant difference between the groups. The HFD+CCl_4_ animals had increased collagen deposition in the liver compared with both SD and HFD groups. Hydroxyproline was also increased in the HFD+CCl_4_ group. Liver enzymes, alanine aminotransferase and aspartate aminotransferase, were increased in the HFD+CCl_4_ group, compared with SD and HFD groups. Also, CCl_4_ was able to trigger an inflammatory process in the liver of HFD-fed animals by promoting an increase of ∼2 times in macrophage activity, ∼6 times in F4/80 gene expression, and pro-inflammatory cytokines (IL-1b and TNFa), in addition to an increase in inflammatory pathway protein phosphorylation (IKKbp). HFD e HFD+CCl_4_ animals increased glucose intolerance compared with SD mice, associated with reduced insulin-stimulated AKT activity in the liver. Therefore, our study has shown that short-term HFD feeding associated with fructose and CCl_4_ can trigger non-alcoholic steatohepatitis and cause damage to glucose metabolism.

## Introduction

Non-alcoholic fatty liver disease (NAFLD) begins with the liver’s accumulation of triacylglycerides (TAG). It is defined as lipid droplets in the cytoplasm of more than 5% of hepatocytes [1], and it is usually associated with hepatic insulin resistance [2,3]. This disease develops when the rate of hepatic TAG synthesis, as a result of increased uptake of fatty acids and their esterification to TAG, as well as *de novo* lipogenesis from carbohydrate and protein metabolism, exceeds the rate of TAG catabolism via fatty acid oxidation or TAG secretion in the form of very low-density lipoproteins (VLDL) [1]. NAFLD represents a broad spectrum of histological abnormalities ranging from simple steatosis to non-alcoholic steatohepatitis (NASH), which may progress to cirrhosis or hepatocellular carcinoma [4]. Long-term fat feeding stimulates the development of fibrosis, a feature highlighted in the NASH [5–7].

In addition to fat feeding, chemical substances induce the fibrotic process such as carbon tetrachloride (CCl_4_) [8]. CCl_4_ is used to study liver fibrosis and cirrhosis in rodents. CCl4 biotransformation depends on CYP2E1 to form the trichloromethyl radical, which is involved in the process of lipid peroxidation [9,10], contributing to necrosis, Kupffer cell activation and inflammatory response [11]. This inflammatory process contributes to the production of several cytokines, which promote the activation of liver stellate cells and, consequently, fibrosis [12].

Studies have shown that a diet rich in fat and enriched with cholesterol (western diet), fructose (23.1 g/L) and glucose (18.9 g/L) in drinking water and treatment with CCl_4_ at the lowest dose (0.2 μl/g) once a week for 12 and 24 weeks, triggered the progression from NASH to extensive fibrosis and cancer in C57BL/6J model [13].

Studies have used various animal models of diets and chemicals to propose models of NASH. However, these models usually go through a long-term diet feeding of approximately 12 weeks with lower doses of CCL_4_ [14]. Therefore, the present study aimed to characterize and propose a new model of NASH, with a short-term developing time (4 weeks), in C57BL/6 mice fed with a high-fat diet supplemented with fructose in the drinking water and treated with CCl_4_.

## Material and methods

### Animals

Male C57BL/6 mice (20–25 g) were kept in a temperature-controlled room at 22 ± 2°C with ad libitum access to food and water, submitted to a 12-h light–dark cycle (light from 6 a.m. to 6 p.m.). These animals were fed with a standard diet (SD) (Nuvilab® - CR-1), consisting of 19% protein, 56% carbohydrate, 3.5% lipids, 5% cellulose and 4.5% vitamins and minerals, providing 3.2 kcal/g of feed; a high-fat diet (HFD) (Research diet - D12079B), consisting of 17% proteins, 43% carbohydrates, and 40% lipids, providing 4.7 kcal/g of feed; and the other group was fed a HFD and supplemented with 10% fructose in the drinking water, and received an intraperitoneal injection of carbon tetrachloride (CCl_4_) (dose: 0.5 μl/g of animal weight) three times a week, the CCl_4_ was diluted in olive oil [8,15]. HFD started when the animals were approximately 8 weeks old. The experiments were carried out after 4 weeks of the onset of HFD. Therefore, we had three groups of experimental animals: animals fed with a standard diet (SD), animals fed only with a high-fat diet (HFD), animals fed with HFD + fructose (10%) and treated with CCl_4_ (HFD+CCl_4_). Ten animals were used per group. The animals were sacrificed under an anesthetic overdose (isoflurane). All animal experiments took place at Ribeirao Preto Medical School at the University of Sao Paulo and all experimental procedures were performed following the animal care principles of the ‘Guidelines for the ethical use of animals in applied etiologies studies’ [16] and previously approved by the FMRP/USP Ethics Committee on Animal Use (No. 1082/2022).

### Body composition

The awake animals were submitted to body composition using the minispec LF50 (Bruker’s, Massachusetts, U.S.A.). Where total body mass, fat mass, lean mass and net weight were analyzed.

### Glucose tolerance test

After 6 h of food restriction, mice were injected intraperitoneally (i.p.) with glucose (1 mg/kg body weight-10% dextrose). Blood samples for measuring glucose were taken by tail bleeding at 0, 15, 30, 45, 60, 90, and 120 min after injection, as previously described [17]. The area under the curve (AUC) was calculated using the statistical software GraphPad Prism 9.0 in order to use it for statistical analysis. Glucose levels were measured in duplicate.

### Lipid and hepatic enzymes measurement

After 6 h of food restriction, the animals were euthanized, and the tissues were removed for lipid content analysis. Tissue TAGs were extracted using the method of Bligh and Dyer [18] and measured using a TAG reagent. The blood of the animals was collected and centrifuged (12000 rpm, 2 min) for analysis of liver enzymes, alanine aminotransferase (ALT), aspartate aminotransferase (AST), plasmatic triglycerides, and cholesterol. The tests were performed using commercial kits (Bioclin, Brazil). The samples were measured in duplicate.

### Lipid peroxidation measurement

Lipid peroxidation was determined by measuring thiobarbituric acid reactive substances (TBARS). Aliquots of 200 μl of samples (blood and tissues) were added to a 400 μl mixture composed of by equal parts of 15% trichloroacetic acid (TCA), 0.25 N HCl and 0.375% TBA, plus 2.5 mM butylated hydroxytoluene (BHT) and 40 μl of 8.1% sodium dodecyl sulfate (SDS), being heated for 30 min at 95°C in an oven. The mixture pH was adjusted to 0.9 with concentrated HCl. BHT was used to prevent lipid peroxidation during heating. After cooling to room temperature and adding 4 ml of butanol, the material was centrifuged at 800 × ***g*** for 15 min at ± 4 °C and the supernatant absorbance was measured at 532 nm. The molar extinction coefficient used was 1.54 × 10^5^ M^−1^ cm^−1^ and the TBARS result was expressed in nmol Eq MDA/ml for plasma and tissue samples [19].

### Hydroxyproline measurement

A liver fragment (80–100 mg) was homogenized in 1 mL hydrolyzed solution. The solution was incubated in a water bath at 95°C for 20 min. After, the pH of the solution was adjusted, pH between 6.0 and 6.8, as described in the commercial kit. Then, the volume was completed with distilled water to a final volume of 10 ml and mixed. After, 3–4 ml of this solution was taken and placed in another tube, and 20–30 mg of acticarbon was added, mixed, and centrifuged at 3500 rpm for 10 min. After this procedure, take 200 μl and pipette into a 96-well plate to carry out the assay protocol described in the commercial kit. The kit used was the Hydroxyproline (Hyp) colorimetric assay kit (Alkali Hydrolysis method) (Elabscience, U.S.A.). The reading was performed at 550 nm on the Accuris SmartReader UV-Vis – Microplate reader. The samples were measured in duplicate.

### Morphological analyzes

#### Oil Red O (ORO) staining

Liver fragments were embedded in a mould with tissue-tek (Thermo Scientific) and frozen in liquid nitrogen (N2). Twelve μm (three sections per slide) were made from different tissue parts. A cryostat (Microm H560) was used to perform the cuts. The slides were stained with ORO (5 min), then washed in running water (30 min). Images (10 of each animal) were obtained using a Nikon Eclipse Ti-U microscope at the 20× objective, with a Nikon DS-Ri1 digital camera and NISElements BR 3.1 software. The quantification of fat accumulation in the tissue was performed using Image J.

#### Sirius red staining

The slides were submitted to Sirius Red staining to identify collagen fibers. Sections were stained with Picrosirius (1 h) and washed in running water (3 min). The red color represented collagen deposition. Ten fields of histological sections (10 images) were analyzed using a 20× objective, taken from three different sections. Collagen was quantified using ImageJ.

### Immunohistochemistry (IHC)

The liver was fixed in a 10% formaldehyde solution for 8 h for immunohistochemistry studies. After dehydrating the samples, the tissue samples were embedded in paraffin at 60°C. Five-micron thick tissue sections were cut transversely on a microtome (Zeiss, Jena, Germany). The tissue was deparaffinized and then hydrated in phosphate buffer (0.2 M) for 10 min. The sections were washed with PBS, incubated with 3% hydrogen peroxide to inhibit endogenous peroxidation for 30 min and then treated with 10% goat serum for 30 min; these procedures were performed at 22°C in a humidified chamber. Next, all the sections were then incubated overnight at 4°C with the primary rabbit antibody F4-80 (Cell Signaling, U.S.A.) diluted in triton X-100 (0.3%) (1:200). The sections were then incubated with the Biotinylated goat anti-polyvalent (Abcam, U.S.A.) for 10 min. Then the sections were washed with PBS and applied Streptavidin peroxidase (Abcam, U.S.A.) was for 10 min and washed with PBS. After, the 3,3-diaminobenzidine (DAB) was utilized as the chromogen, yielding the overall brown colour. Negative controls were conducted for each antibody by omitting the primary antibody. At least two samples from each animal were independently analyzed. Images were acquired with a Nikon DS-R1 digital camera connected to a Nikon Eclipse Ti-U microscope. The immunostained sections were quantified by using the ImageJ software. At least 5 areas per slide were selected and photographed blinded. The expression of F4/80 was calculated as follows: the total area of the liver (specimen) was outlined, and the area occupied by cells expressing F4/80 was quantified using the image analyzer within the reference area. A color for F4/80 was pre-defined and applied to the selected area. The result was expressed as a percentage of the positive area concerning the total area.

### Protein expression

Animals were deeply anesthetized with isoflurane (5%), and after the loss of corneal reflexes, the abdominal wall was opened, and the liver was removed. After that, pieces of the liver (∼50 mg) were homogenized in RIPA buffer with protease and phosphatase inhibitor cocktail (TermoFisher, U.S.A.). The tissue extracts were centrifuged at 15000 ***g***, at 4°C, for 20 min. The protein content of the supernatants was measured by the Bradford method. Aliquots of the supernatant, containing 50 μg total protein, were treated with Novex tris-glycine SDS buffer and RIPA buffer (TermoFisher, U.S.A.), loaded onto Novex WedgeWell 4% and 20% tris-glycine gel (TermoFisher, U.S.A.) and subjected to SDS-PAGE. The proteins were transferred from the gels to nitrocellulose membranes using a Bio-Rad Trans-Blot Semi-Dry (U.S.A.). The membranes were incubated in TBST-B blocking buffer (10 mM Tris, 150 mM NaCl, 0.05% tween 20, and 5% skim milk) for 2 h at RT to prevent nonspecific binding to the membrane. The nitrocellulose membranes were incubated with the specific primary antibodies overnight at 4°C and subsequently incubated with a secondary antibody conjugated to horseradish peroxidase for 1 h at RT. The immunoblots were developed using the SuperSignal® West Pico Chemiluminescent Substrate (BioRad, U.S.A.). The immunoblots were visualized using an iBright 750 (TermoFisher, U.S.A.) and quantified using the ImageJ software (imagej.net/Downloads). The primary antibodies used were: pNFkB (catalogue number 3033 S, Cell Signaling), pIkkβ (catalogue number 2694 S, Cell Signaling), Ikkβ (catalogue number sc-8014, Santa Cruz), pJNK (catalogue number 9255 S, Cell Signaling), JNK (catalogue number 9252S, Cell Signaling), pAKTser474 (catalogue number 8599 S, Cell Signaling), AKT (catalogue number 126811, Abcam), GAPDH (catalogue number 2118 S).

### Gene expression

Total RNA from the liver was extracted with a Total RNA purification kit (Cellco, Brazil) and reverse transcribed into cDNA (High-Capacity cDNA kit, Applied Biosystems, Waltham, MA, USA). Gene expression was evaluated by RT-PCR using Rotor-Gene Q (Qiagen, Hilden, Germany) and SYBR Green as the fluorescent dye (Platinum® SYBR® Green qPCR Supermix UDG, Invitrogen, Waltham, MA, U.S.A.). The gene expression analysis was carried out using a method previously described [20,21]. The samples were measured in duplicate. The primers used are described in Table 1.

**Table 1:** Primer sequences

Primers	Forward	Reverse
TNFα	TCTTCTCATTCCTGCTTGTGGC	CACTTGGTGGTTTGCTACGACG
IL-1β	GGCAGCTACCTGTGTCTTTCCC	ATATGGGTCCGACAGCACGAG
F4/80	CCTGGACGAATCCTGTGAAG	GGTGGGACCACAGAGAGTTG
TGF-β	CTCCCGTGGCTTCTAGTGC	GCCTTAGTTTGGACAGGATCTG
Col1α	GCTCCTCTTAGGGGCCACT	CCACGTCTCACCATTGGGG
MMP2	GCCCCCATGAAGCCTTGTTT	GAACTTGCAGGGCTGTCCAT

### Statistical analysis

The results were analyzed using the GraphPad Prism version 9.0® program (GraphPad Software, La Jolla, CA, U.S.A.). The minimum sample size per group for each parameter analyzed was defined by an n sufficient to analyze the distribution of samples through the ‘D′Agostino and Pearson omnibus normality test’ recommended by the GraphPad Prism version 9.0® program. All samples were evaluated for normal distribution and subjected to either a one-way ANOVA followed by the post-hoc Bonferroni test (Bonferroni Multiple Comparison Test) (*P*<0.05). The results were expressed as mean ± standard error of the mean (mean ± SEM).

## Results

### Effect of CCl_4_ on the accumulation of lipids in animals fed a high-fat diet

**Figure 1:**
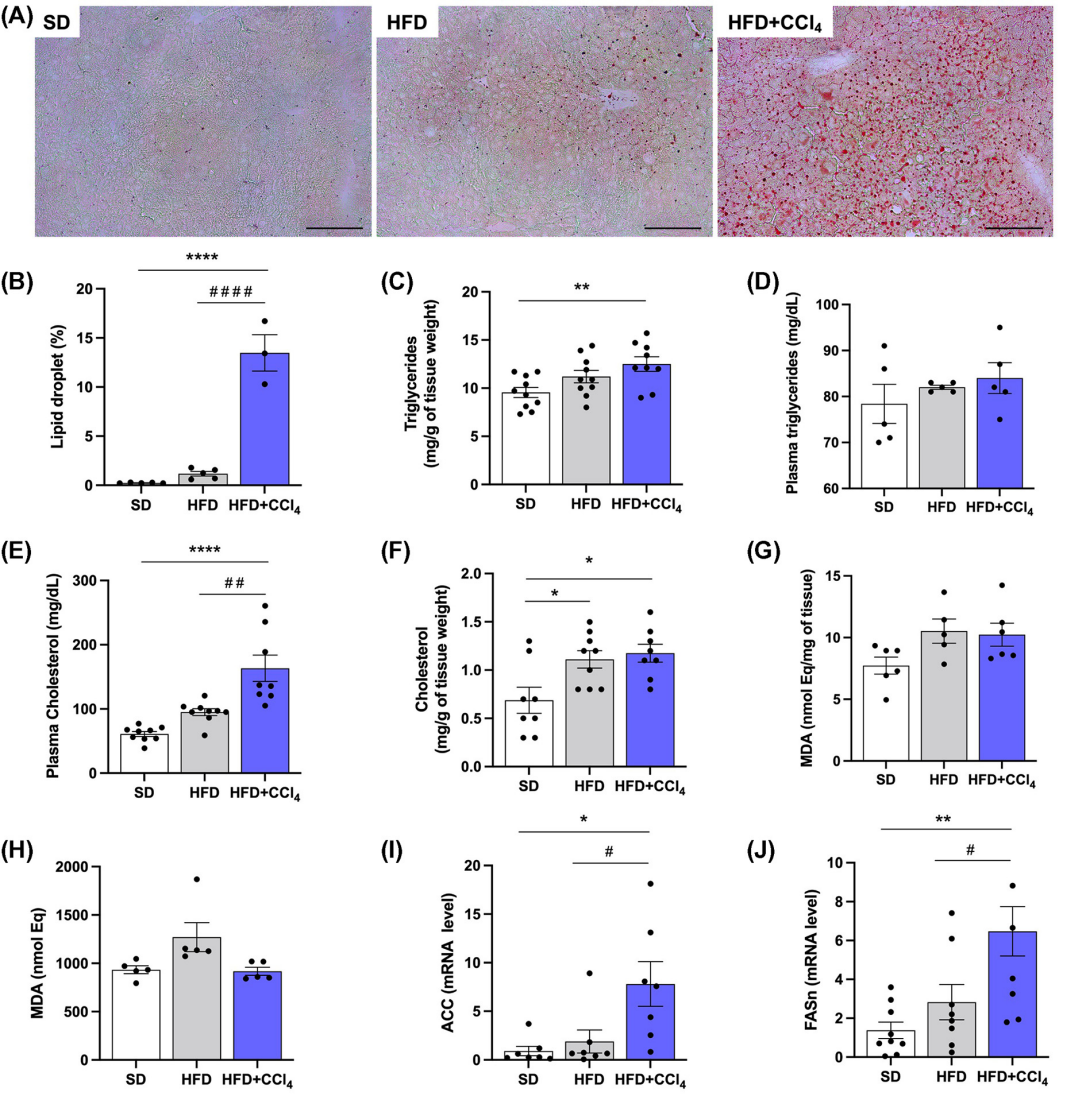
Carbon tetrachloride promotes increased hepatic steatosis in animals fed a high-fat diet (**A**) Representative images of fat droplets in the liver. Histological sections stained with Oil red O stain. (**B**) Quantification of lipid droplets. (**C**) Triglycerides measurement in liver tissue. (**D**) Measurement of plasma triglycerides. (**E**) Plasma cholesterol measurement. (**F**) Tissue cholesterol measurement. (**G**) Tissue malondialdehyde (MDA) measurement. (**H**) Plasma malondialdehyde (MDA) measurement. (**I**) Gene expression of Acetyl-CoA carboxylase (ACC). (**J**) Gene expression of fatty acid synthase (FASN). The black bar in the images represents a scale of 100 μm and objective 20×. Columns and vertical bars represent mean and e.p.m., respectively. One-way ANOVA followed by Bonferroni post-test, *P<0.05, **P<0.01 and ****P<0.0001 (compared with SD), ^#^*P*<0.05, ^# #^*P*<0.01 and ^####^*P*<0.0001 (compared with HFD) (quantification of lipid droplets by ORO, *n*=4–5; Tissue triglycerides and cholesterol, *n*=9; Plasma triglycerides and cholesterol, *n*=5; Plasma MDA, *n*=5; Tissue MDA, *n*=5–6; Gene expression, *n*=7–9).

After feeding a high-fat diet and injecting CCl_4_ for 4 weeks, the animals were euthanized, and the liver and plasma were collected for analysis. According to the morphological parameters, the animals fed with HFD+CCl_4_ presented hepatic steatosis, as can be seen in the image below (Figure 1A), and in the quantification of fat droplets in these images, with an increase in this lipid deposition of ∼13%, when compared with animals fed a standard diet (0.2%) and HFD (1.2%) group. The HFD and SD groups had not significant difference (Figure 1B). Tissue triglyceride measurement was also increased in the HFD+CCl_4_ group (12.5 ± 0.8 mg/g of tissue weight) when compared to the SD group (9.5 ± 0.5 mg/g of tissue weight) and HFD (11.2 ± 0.6 mg/g of tissue weight), without significant difference between the SD and HFD groups (Figure 1C). Plasma triglycerides showed no significant difference between groups (SD: 78.4 ± 4.2, HFD: 82.0 ± 0.4, HFD+CCl_4_: 84.0 ± 3.3 mg/dL) (Figure 1D). Plasma cholesterol was increased in animals from the HFD+CCl_4_ group (163.3 ± 20.5 mg/dL) when compared with the SD group (61.1 ± 3.9 mg/dL) and HFD (95.0 ± 5.5 mg/dL), without significant difference between the SD and HFD groups (Figure 1E). Tissue cholesterol measurement was increased in 57% in both HFD and HFD+CCl_4_ groups, when compared with SD (Figure 1F). The increase of lipids in the tissue can promote lipid peroxidation, so we evaluated lipid peroxidation markers such as malondialdehyde (MDA). However, we observed no significant difference in MDA in either plasma or tissue between the groups (Figure 1G,H). We also evaluated the gene expression of enzymes related to *de novo* lipogenesis, such as Acetyl-CoA carboxylase (ACC) and Fatty acid synthase (FASN), and observed an increase by 9- and 5-fold, respectively, in the HFD+CCL_4_ group compared with the SD and HFD groups (Figure 1I,J). Thus, the high-fat diet with fructose and CCl_4_ promotes hepatic steatosis.

### Effect of CCl_4_ in the hepatic inflammation in animals fed a high-fat diet

**Figure 2:**
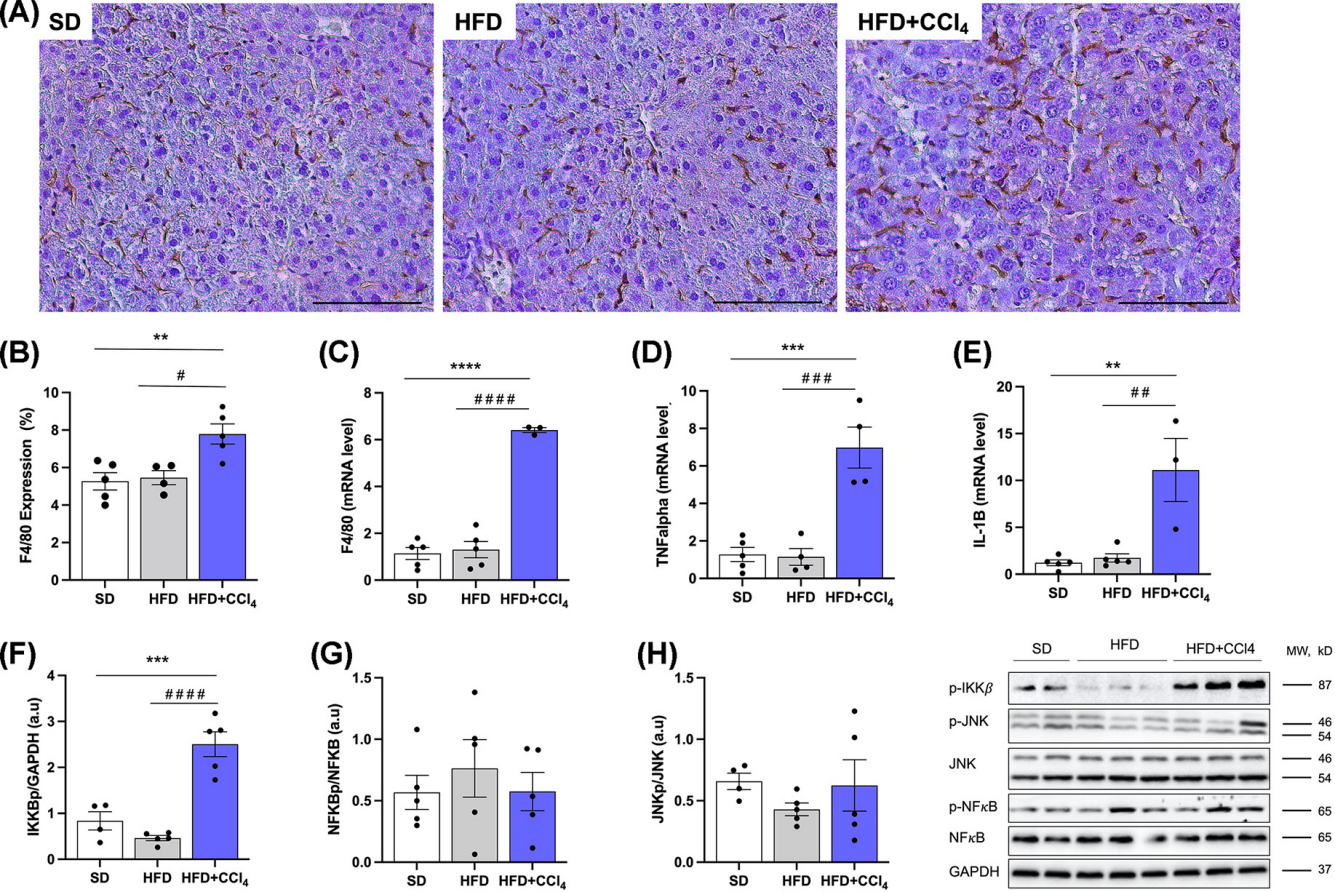
Carbon tetrachloride promotes inflammation in the liver of animals fed a high-fat diet (**A**) Representative images of macrophage marker. Histological sections stained with F4/80 by immunohistochemistry. (**B**) Quantification of F4/80. (**C–E**) Gene expression of pro-inflammatory cytokines. (**F–H**) Expression of proteins of the inflammatory pathway. The black bar in the images represents a scale of 100 μm and objective 20×. Columns and vertical bars represent mean and e.p.m., respectively. One-way ANOVA followed by Bonferroni post-test, **P<0.01, ***P<0.001 and ****P<0.0001 (compared with SD), ^#^*P*<0.05; ^##^*P*<0.01; ^###^*P*<0.001; ^####^*P*<0.0001 (compared with HFD) (F4/80 quantification, *n*=4–5; Gene expression, *n*=3–5; Protein expression, *n*=4–5).

CCl_4_ showed that it triggers an inflammatory process in the liver of animals fed a high-fat diet by promoting an increase of approximately ∼2 times in macrophage activity, as can be seen in the image below and its quantification (Figure 2A,B), in addition to the increase of 6 times in the gene expression of the macrophage marker F4/80, and pro-inflammatory cytokines, 9 times for IL-1b and 5 times for TNFa (Figure 2C–E). In addition to the 190% increase in inflammatory pathway protein phosphorylation (IKKbp) (Figure 2F), these data were compared with the SD and HFD groups. JNK and NFkB activity did not differ significantly between groups. Thus, treatment with CCl_4_ has been shown to stimulate the inflammatory process in the liver.

### Effect of CCl_4_ on collagen deposition in animals fed a high-fat diet

**Figure 3:**
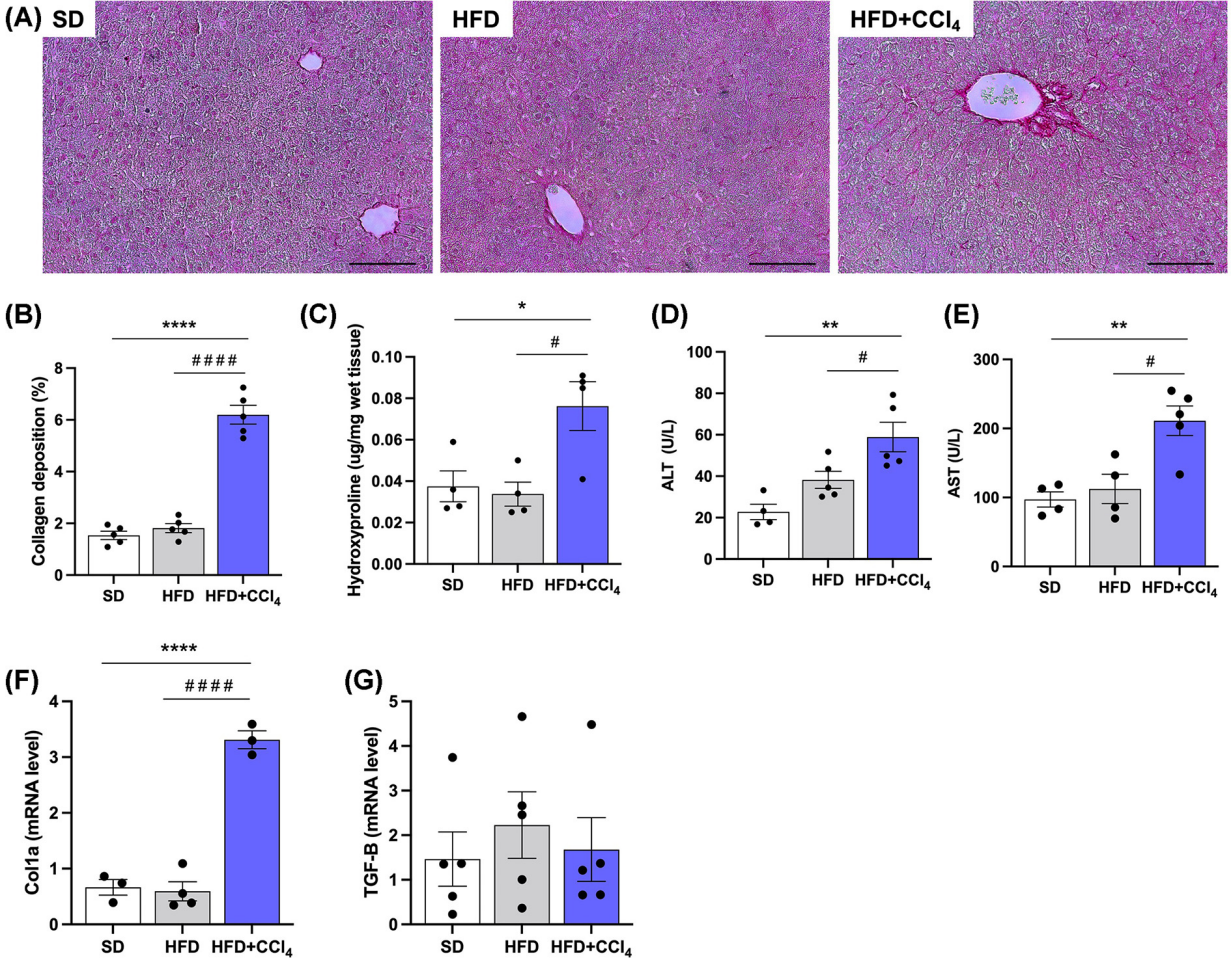
Carbon tetrachloride promotes increase of collagen in the liver of animals fed a high-fat diet Representative images of collagen deposition in the liver. (**A**) Histological sections stained with Sirius Red. (**B**) Collagen quantification. (**C**) Hydroxyproline measurement in liver tissue. (**D**) Alanine aminotransferase (ALT) measurement in plasma. (**E**) Plasma aspartate aminotransferase (AST) measurement. (**F,G**). Genes expression that encode proteins that participate in collagen production. The black bar in the images represents a scale of 100 μm and objective 20×. Columns and vertical bars represent mean and e.p.m., respectively. One-way ANOVA followed by Bonferroni post-test, *P<0.05; **P<0.01; ****P<0.0001 (compared with SD), ^#^*P*<0.05; ^####^*P*<0.0001 (compared with HFD) (Collagen quantification by sirius red, *n*=5; hydroxyproline in tissue, *n*=4; Plasma levels of ALT and AST, *n*=5; Gene expression, *n*=3–5).

Parameters related to liver fibrosis were analyzed, and it was seen that the animals fed with HFD and treated with CCl_4_ had an increase of 4-fold in the liver collagen deposition compared with the SD and HFD groups. These results demonstrated an increase in collagen deposition in the HFD+CCl_4_ group, as seen in the figure below and the quantification of collagen through the images (Figures 3A,B). In addition, hydroxyproline, an amino acid that is in the composition of the collagen, was measured, and there was also an increase of ∼130% in this parameter in the HFD+CCl_4_ group when compared with the SD and HFD groups, which did not have a significant difference between them (Figure 3C). Liver enzymes were analyzed, alanine aminotransferase (ALT) and aspartate aminotransferase (AST), which were increased in the HFD+CCl_4_ group (58.9 ± 7.1 U/L; 211.2 ± 21.7 U/L, respectively), compared with the SD group (22.7 ± 3.7 U/L; 97.1 ± 11.0 U/L, respectively) and the HFD group (38.2 ± 4.1 U/L; 112.4 ± 21.3 U/L, respectively), both had no significant difference between HFD and HFD+CCl_4_ (Figure 3D,E). In addition, the expression of some genes that encode proteins related to collagen production, such as TGF-b and collagen 1a (col1a), was demonstrated. Col1a expression was increased 5 times in the HFD+CCl_4_ group compared with the SD and HFD groups, which did not show a significant difference between them. At the same time, the gene expression of TGF-b showed no significant difference between groups (Figure 3F,G). These results show that the high-fat diet with fructose and CCl_4_ stimulates steatohepatitis’s progression since liver collagen deposition was increased.

### Effect of CCl_4_ in the body mass and glucose metabolism in animals fed a high-fat diet

**Figure 4:**
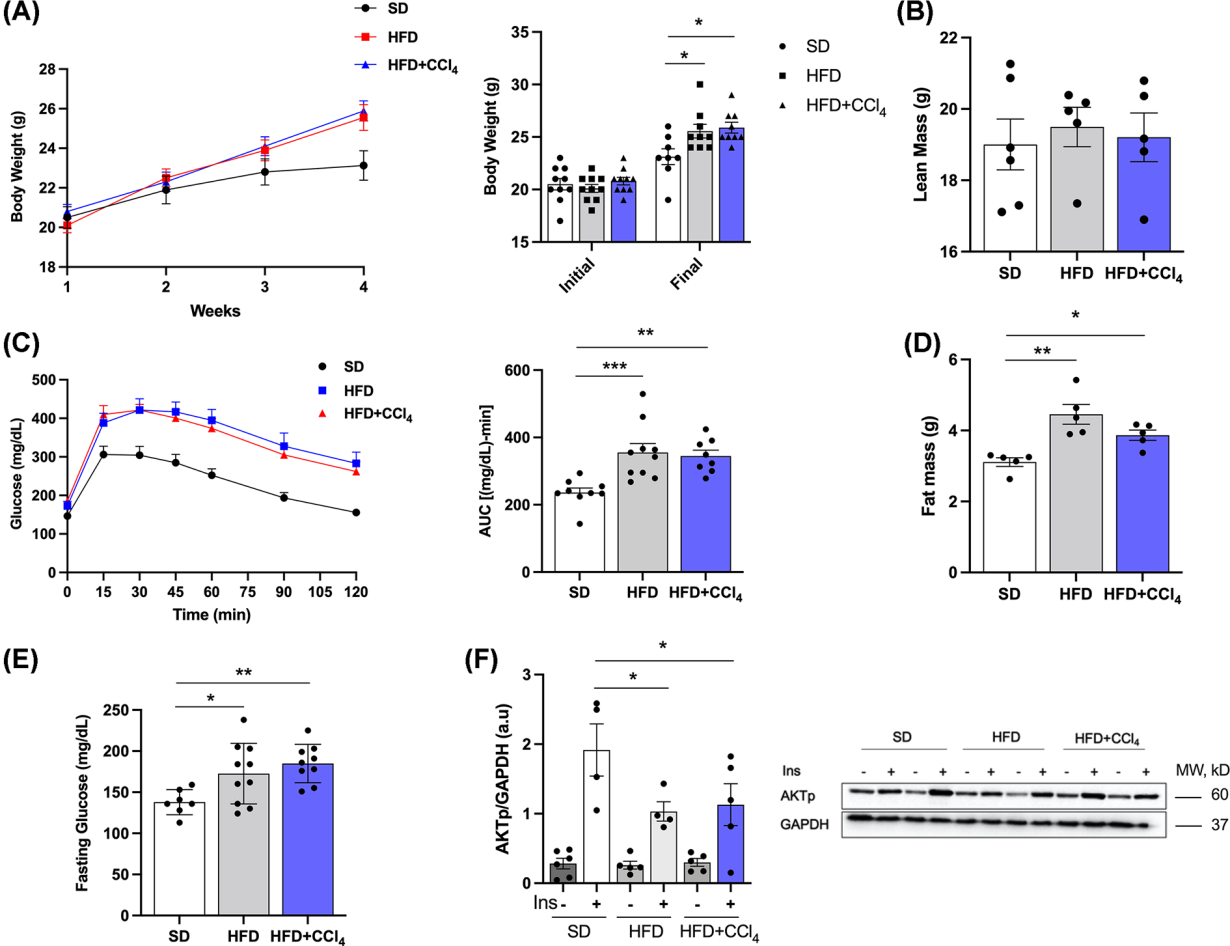
High-fat diet (HFD) promotes increased body mass and glucose intolerance (**A**) Total body mass. (**B**) Lean mass. (**C**) Glucose tolerance test (GTT) and area under the curve (AUC). (**D**) Fat mass. (**E**) Fasting glucose. (**F**) Phosphorylation of AKT (insulin signaling pathway protein). Columns and vertical bars represent mean and e.p.m., respectively. One-way ANOVA followed by Bonferroni post-test, *P<0.05; **P<0.01; ***P<0.001 (compared with SD) (body mass, *n*=8–9; lean mass and fat: 5–6, GTT and fasting blood glucose, *n*=8–10; Phosphorylated protein: *n* = 4–5); Ins, insulin stimulation.

The total body mass of the animals increased with the high-fat diet (25.6 ± 0.6 g) and in the HFD animals treated with CC_l4_ (25.9 ± 0.5 g) when compared with the SD (23.1 ± 0.7 g) (Figure 4A), and there was no significant difference between the HFD and HFD+CCl_4_ groups. Similar results were obtained concerning fat mass (SD: 3.1 ± 0.1 g; HFD: 4.4 ± 0.3 g; HFD+CCl_4_: 3.9 ± 0.1 g). No significant difference was observed in the lean mass of the animals between the groups (Figures 4B,C). After 4 weeks of a high-fat diet and treatment with CCl_4_, the animals were submitted to a glucose tolerance test (GTT). It was seen that both animal groups fed with HFD and HFD treated with CCl_4_ were glucose intolerant, maintaining increased values during the entire glycemic curve, with values for the area under the curve of 355.5 ± 26.3 mg/dL-min and 344.7 ± 17.6 mg/dL-min, respectively, when compared with the control SD (236.2 ± 13.7 mg/dL-min) (Figure 4D). Fasting glucose increased in both the HFD (172.6 + 11.7 mg/dL) and HFD+CCL_4_ (184.9 + 7.8 mg/dL) groups when compared to the SD (138.0 + 5.8 mg/dL) (Figure 4E). Furthermore, a reduction in the activity of AKT, a protein of the insulin signaling pathway, was observed in both HFD (46%) and HFD+CCl_4_ (41%) groups compared with the SD group (Figure 4F). Thus, the high-fat diet promotes glucose intolerance and impaired insulin signaling, results that are maintained with treatment with CCl_4_.

## Discussion

We investigated the effect of CCl4 toxin and a high-fat diet enriched with fructose on steatohepatitis and glucose metabolism parameters. We observed that CCl_4_, coupled with a high-fat diet and fructose, stimulates an increase in hepatic triglycerides and plasmatic cholesterol, as well as collagen deposition in the liver and hepatic transaminases in the plasma. In addition, we saw an increase in macrophage activity, proteins and cytokines pro-inflammatory. Moreover, the high-fat diet was able to promote glucose intolerance and decrease the protein activity of the insulin pathway.

CCl_4_ applied orally can lead to increased liver weight, lipids accumulation, liver enzyme activity, inflammation, damage and cell death, which leads to the development of fibrosis, cirrhosis and hepatocellular carcinoma if this oral exposure is long-term [22,23]. The isolated application of CCl_4_ (dose: 0.5–0.7 ml/g body weight), without the presence of a high-fat diet, twice a week and for 6 weeks, or three times a week for four weeks, demonstrated the deposition of collagen in the liver, which was already characterized as fibrosis. However, this work did not characterize lipid deposition [23].

Animal model fed only with a high-fat diet promotes hepatic steatosis after 12 weeks of diet [24]. Our results have shown that a high-fat and fructose-enriched diet for only 4 weeks, associated with intraperitoneal injections of CCl_4_ three times a week during the same period as the diet, promotes an increase in the deposition of lipids in the liver. Fructose has been associated with non-alcoholic fatty liver disease, and this has been confirmed in different studies [25–27]. It is not fructose itself that causes the accumulation of triglycerides. The increase of fructose promotes the activation of *de novo* lipogenesis and the blocks of the fatty acid oxidation [28]. In our study, we demonstrated an increase in enzymes related to *de novo* lipogenesis, such as ACC and FASN, in the group that received fructose, which was also the group in which CCl_4_ was administered.

In addition, we showed an increase in collagen deposition in the liver, both by quantification of collagen by images and by measuring hydroxyproline in the liver, an amino acid that makes up collagen [29], associated with an increase in gene expression 1 alpha collagen in the tissue. These data corroborate results previously published [23]. Other studies have described that HFD with CCl_4_ administration has promoted inflammation and apoptosis, leading to fibrosis in mice. In one of these studies, the animals were fed for 12 weeks with HFD, and in the last 4 weeks, they were treated with CCl4 twice a week (dose: 0.05–0.1 ml/kg) [14]. In another study, the animals were fed a Western diet, a high-fat diet enriched with cholesterol, for 12 and 24 weeks, with simultaneous administration of CCl_4_ once a week at doses of 0.32 mg/g. Also, they presented steatosis and fibrosis [13]. Concerning our study, we reduced the time on a high-fat diet and increased the dose (0.5 ml/g) and the number of applications per week of CCl_4_ (three times a week) in the animals, and we obtained similar results.

Non-alcoholic fatty liver disease (NAFLD) is classified into two subtypes, non-alcoholic fatty liver (NAFL) and non-alcoholic steatohepatitis (NASH), the progressive form of the disease. NASH is characterized by steatosis, lobular inflammation and ballooning of hepatocytes, with or without fibrosis [30]. A previously described mechanism that leads to NAFLD is related to hepatic energy imbalance, where the increased energy intake by an elevated consumption of carbohydrates and lipids exceeds the oxidation of this energy in CO2 or exports it as very-low-density lipoproteins (VLDLs). This imbalance promotes the accumulation of lipids as TAG in the liver [31–34]. The accumulation of lipids in the liver is toxic, generating what is known as lipotoxicity, as well as CCl_4_ itself, which is a hepatotoxin. This lipotoxicity disrupts normal cellular processes, promoting cell damage and death, leading to inflammatory processes and the replacement of functional cells by fibroblasts and extracellular matrix [35].

Our model presented hepatic steatosis and fibrosis, as previously described, and an inflammatory condition, which we demonstrated through of increase in the macrophage activity by the F4/80 marker, by the increase in the gene expression of F4/80 and pro-cytokines (IL-1B and TNF-α), in addition to increased activity of the IKKB protein, proteins of the inflammatory pathway. From this set of results, our model represents a model of steatohepatitis. In our laboratory, another NASH model was established using ApoE knockout mice and fed with different diets, HFD and western diet, both groups showed similar results to those described in this study, such as imbalance in glucose metabolism, hepatic steatosis and inflammation, as well as increased collagen deposition in the liver when compared with the group fed the standard diet [6].

Type 2 diabetes is considered a risk factor for the progression of NASH, increasing the progression of fibrosis and subsequently may lead to cirrhosis, promoting increased mortality from liver diseases. Like obesity and dyslipidemia, however, diabetes is still a significant risk factor [36]. Given this strong relationship between Type 2 diabetes and NASH, we evaluated some parameters related to glucose metabolism, and we saw that the high-fat diet promoted an increase in the body mass of the animals and fat mass. In addition, we observed glucose intolerance, fasting glucose increased and damage to the insulin signaling pathway, which may indicate insulin resistance. A high-fat diet promotes insulin resistance in adipose tissue, which leads to hyperglycemia and lipotoxicity due to excess of lipolysis, as insulin plays a crucial role in glucose uptake and inhibition of lipolysis in adipose tissue [3,37,38]. In addition to the adipose tissue, insulin acts on the liver, inhibiting gluconeogenesis and glycogenolysis. Once there is a change in glucose metabolism, such as insulin resistance triggered by HFD, whether, in patients or an animal model, the liver will produce more glucose, which leads to increased plasma glucose [35,37].

## Conclusion

Therefore, our study has shown that a high-fat diet enriched with fructose in the short term, coupled with carbon tetrachloride, can trigger NASH, increased adiposity and impaired glucose metabolism.

## Data Availability

All original raw data is available at the time of submission. As per the Data Policy, this data will be stored for a minimum of 10 years and will be made available to the Editorial Office, Editors and readers upon request.

## Abbreviations

ALT: alanine aminotransferase
AST: aspartate aminotransferase
AUC: area under the curve
CCl_4_: carbon tetrachloride
GTT: glucose tolerance test
HFD: high-fat diet
Hyp: hydroxyproline
NAFLD: non-alcoholic fatty liver disease
NASH: non-alcoholic steatohepatitis
ORO: Oil Red O
SD: standard diet
TAG: triacylglycerides
VLDL: very low-density lipoprotein

## References

[1] Birkenfeld A.L. and Shulman G.I. (2014) Nonalcoholic fatty liver disease, hepatic insulin resistance, and type 2 diabetes. Hepatology 59, 713–723 10.1002/hep.26672 23929732 PMC3946772

[2] Camporez J.P.G., Kanda S., Petersen M.C., Jornayvaz F.R., Samuel V.T., Bhanot S. et al. (2015) ApoA5 knockdown improves whole-body insulin sensitivity in high-fat-fed mice by reducing ectopic lipid content. J. Lipid Res. 56, 526–536 10.1194/jlr.M054080 25548259 PMC4340301

[3] Camporez J.P., Lyu K., Goldberg E.L., Zhang D., Cline G.W., Jurczak M.J. et al. (2019) Anti-inflammatory effects of oestrogen mediate the sexual dimorphic response to lipid-induced insulin resistance. J. Physiol. 597, 3885–3903 10.1113/JP277270 31206703 PMC6876753

[4] Ipsen D.H., Lykkesfeldt J. and Tveden-Nyborg P. (2018) Molecular mechanisms of hepatic lipid accumulation in non-alcoholic fatty liver disease. Cell. Mol. Life Sci. 75, 3313–3327 10.1007/s00018-018-2860-6 29936596 PMC6105174

[5] Araujo L.C.C., Cruz A.G., Camargo F.N., Sucupira F.G., Moreira G.V., Matos S.L. et al. (2023) Estradiol protects female ApoE KO mice against western-diet-induced non-alcoholic steatohepatitis. Int. J. Mol. Sci. 24, 9845 10.3390/ijms24129845 37372993 PMC10298710

[6] Camargo F.N., Matos S.L., Araujo L.C.C., Carvalho C.R.O., Amaral A.G. and Camporez J.P. (2022) Western diet-fed ApoE knockout male mice as an experimental model of non-alcoholic steatohepatitis. Curr. Issues Mol. Biol. 44, 4692–4703 10.3390/cimb44100320 36286035 PMC9600038

[7] Asgharpour A., Cazanave S.C., Pacana T., Seneshaw M., Vincent R., Banini B.A. et al. (2016) A diet-induced animal model of non-alcoholic fatty liver disease and hepatocellular cancer. J. Hepatol. 65, 579–588 10.1016/j.jhep.2016.05.005 27261415 PMC5012902

[8] Yanguas S.C., Cogliati B., Willebrords J., Maes M., Colle I., van den Bossche B. et al. (2016) Experimental models of liver fibrosis. Arch. Toxicol. 90, 1025–1048 10.1007/s00204-015-1543-4 26047667 PMC4705434

[9] Basu S. (2003) Carbon tetrachloride-induced lipid peroxidation: eicosanoid formation and their regulation by antioxidant nutrients. Toxicology 189, 113–127 10.1016/S0300-483X(03)00157-4 12821287

[10] Weber L.W., Boll M. and Stampfl A. (2003) Hepatotoxicity and mechanism of action of haloalkanes: carbon tetrachloride as a toxicological model. Crit. Rev. Toxicol. 33, 105–136 10.1080/713611034 12708612

[11] Heindryckx F., Colle I. and Van Vlierberghe H. (2009) Experimental mouse models for hepatocellular carcinoma research. Int. J. Exp. Pathol. 90, 367–386 10.1111/j.1365-2613.2009.00656.x 19659896 PMC2741148

[12] Iwaisako K., Jiang C., Zhang M., Cong M., Moore-Morris T.J., Park T.J. et al. (2014) Origin of myofibroblasts in the fibrotic liver in mice. Proc. Natl. Acad. Sci. U.S.A. 111, E3297–E3305 10.1073/pnas.1400062111 25074909 PMC4136601

[13] Tsuchida T., Lee Y.A., Fujiwara N., Ybanez M., Allen B., Martins S. et al. (2018) A simple diet- and chemical-induced murine NASH model with rapid progression of steatohepatitis, fibrosis and liver cancer. J. Hepatol. 69, 385–395 10.1016/j.jhep.2018.03.011 29572095 PMC6054570

[14] Kubota N., Kado S., Kano M., Masuoka N., Nagata Y., Kobayashi T. et al. (2013) A high-fat diet and multiple administration of carbon tetrachloride induces liver injury and pathological features associated with non-alcoholic steatohepatitis in mice. Clin. Exp. Pharmacol. Physiol. 40, 422–430 10.1111/1440-1681.12102 23611112

[15] Tan H., He Q., Li R., Lei F. and Lei X. (2016) Trillin reduces liver chronic inflammation and fibrosis in carbon tetrachloride (CCl4) induced liver injury in mice. Immunol. Invest. 45, 371–382 10.3109/08820139.2015.1137935 27219527

[16] Sherwin C.M., Christiansen S.B., Duncan I.J., Erhard H.W., Lay D.C., Mench J.A. et al. (2003) Guidelines for the ethical use of animals in applied ethology studies. Appl. Anim. Behav. Sci. 81, 291–305 10.1016/S0168-1591(02)00288-5

[17] Talarico C.H.Z., Alves E.S., Dos Santos J.D.M., Sucupira F.G.S., Araujo L.C.C. and Camporez J.P. (2023) Progesterone Has No Impact on the Beneficial Effects of Estradiol Treatment in High-Fat-Fed Ovariectomized Mice. Curr. Issues Mol. Biol. 45, 3965–3976 10.3390/cimb45050253 37232722 PMC10216949

[18] Bligh E.G. and Dyer W.J. (1959) A rapid method of total lipid extraction and purification. Can J. Biochem. Physiol. 37, 911–917 10.1139/y59-099 13671378

[19] Lapenna D., Ciofani G., Pierdomenico S.D., Giamberardino M.A. and Cuccurullo F. (2001) Reaction conditions affecting the relationship between thiobarbituric acid reactivity and lipid peroxides in human plasma. Free Radic. Biol. Med. 31, 331–335 10.1016/S0891-5849(01)00584-6 11461770

[20] Livak K.J. and Schmittgen T.D. (2001) Analysis of relative gene expression data using real-time quantitative PCR and the 2(-Delta Delta C(T)) Method. Methods 25, 402–408 10.1006/meth.2001.1262 11846609

[21] Pfaffl M.W. (2001) A new mathematical model for relative quantification in real-time RT-PCR. Nucleic Acids Res. 29, e45 10.1093/nar/29.9.e45 11328886 PMC55695

[22] Korsrud G.O., Grice H.C. and McLaughlan J.M. (1972) Sensitivity of several serum enzymes in detecting carbon tetrachloride-induced liver damage in rats. Toxicol. Appl. Pharmacol. 22, 474–483 10.1016/0041-008X(72)90255-4 5041376

[23] Scholten D., Trebicka J., Liedtke C. and Weiskirchen R. (2015) The carbon tetrachloride model in mice. Lab. Anim. 49, 4–11 10.1177/0023677215571192 25835733

[24] Araujo L.C.C., Feitosa K.B., Murata G.M., Furigo I.C., Teixeira S.A., Lucena C.F. et al. (2018) Uncaria tomentosa improves insulin sensitivity and inflammation in experimental NAFLD. Sci. Rep. 8, 11013 10.1038/s41598-018-29044-y 30030460 PMC6054645

[25] Ouyang X., Cirillo P., Sautin Y., McCall S., Bruchette J.L., Diehl A.M. et al. (2008) Fructose consumption as a risk factor for non-alcoholic fatty liver disease. J. Hepatol. 48, 993–999 10.1016/j.jhep.2008.02.011 18395287 PMC2423467

[26] Lim J.S., Mietus-Snyder M., Valente A., Schwarz J.M. and Lustig R.H. (2010) The role of fructose in the pathogenesis of NAFLD and the metabolic syndrome. Nat. Rev. Gastroenterol. Hepatol. 7, 251–264 10.1038/nrgastro.2010.41 20368739

[27] Vos M.B. and Lavine J.E. (2013) Dietary fructose in nonalcoholic fatty liver disease. Hepatology 57, 2525–2531 10.1002/hep.26299 23390127

[28] Jensen T., Abdelmalek M.F., Sullivan S., Nadeau K.J., Green M., Roncal C. et al. (2018) Fructose and sugar: A major mediator of non-alcoholic fatty liver disease. J. Hepatol. 68, 1063–1075 10.1016/j.jhep.2018.01.019 29408694 PMC5893377

[29] Li P. and Wu G. (2018) Roles of dietary glycine, proline, and hydroxyproline in collagen synthesis and animal growth. Amino Acids 50, 29–38 10.1007/s00726-017-2490-6 28929384

[30] Loomba R., Friedman S.L. and Shulman G.I. (2021) Mechanisms and disease consequences of nonalcoholic fatty liver disease. Cell 184, 2537–2564 10.1016/j.cell.2021.04.015 33989548 PMC12168897

[31] Petersen K.F. and Shulman G.I. (2002) Pathogenesis of skeletal muscle insulin resistance in type 2 diabetes mellitus. Am. J. Cardiol. 90, 11G–18G 10.1016/S0002-9149(02)02554-7 12231074

[32] Shulman G.I. (2000) Cellular mechanisms of insulin resistance. J. Clin. Invest. 106, 171–176 10.1172/JCI10583 10903330 PMC314317

[33] Camporez J.P., Wang Y., Faarkrog K., Chukijrungroat N., Petersen K.F. and Shulman G.I. (2017) Mechanism by which arylamine N-acetyltransferase 1 ablation causes insulin resistance in mice. Proc. Natl. Acad. Sci. U.S.A. 114, E11285–E11292 10.1073/pnas.1716990115 29237750 PMC5748223

[34] Wehmeyer M.H., Zyriax B.C., Jagemann B., Roth E., Windler E., Schulze Zur Wiesch J. et al. (2016) Nonalcoholic fatty liver disease is associated with excessive calorie intake rather than a distinctive dietary pattern. Medicine (Baltimore). 95, e3887 10.1097/MD.0000000000003887 27281105 PMC4907683

[35] Heydemann A. (2016) An overview of murine high fat diet as a model for type 2 diabetes mellitus. J. Diabetes Res. 2016, 2902351 10.1155/2016/2902351 27547764 PMC4983380

[36] Powell E.E., Wong V.W. and Rinella M. (2021) Non-alcoholic fatty liver disease. Lancet 397, 2212–2224 10.1016/S0140-6736(20)32511-3 33894145

[37] Petersen M.C. and Shulman G.I. (2018) Mechanisms of insulin action and insulin resistance. Physiol. Rev. 98, 2133–2223 10.1152/physrev.00063.2017 30067154 PMC6170977

[38] Guo F., Ma Y., Kadegowda A.K.G., Betters J.L., Xie P., Liu G. et al. (2013) Deficiency of liver comparative gene identification-58 causes steatohepatitis and fibrosis in mice. J. Lipid Res. 54, 2109–2120 10.1194/jlr.M035519 23733885 PMC3708361

